# Comparison of Intact Fish Skin Graft and Allograft as Temporary Coverage for Full-Thickness Burns: A Non-Inferiority Study

**DOI:** 10.3390/biomedicines12030680

**Published:** 2024-03-18

**Authors:** Randolph Stone, Emily C. Saathoff, David A. Larson, John T. Wall, Nathan A. Wienandt, Skuli Magnusson, Hilmar Kjartansson, Robert J. Christy, Shanmugasundaram Natesan

**Affiliations:** 1 Combat Wound Care Research Department, US Army Institute of Surgical Research, JBSA Fort Sam Houston, Houston, TX 78234, USA; emilycsaathoff@gmail.com (E.C.S.); davidlarson15@gmail.com (D.A.L.); johntwall4@gmail.com (J.T.W.); christyr1@uthscsa.edu (R.J.C.); shansnatesan@gmail.com (S.N.); 2 Comparative Pathology Department, US Army Institute of Surgical Research, JBSA Fort Sam Houston, Houston, TX 78234, USA; nwienandtdvmdacvp@gmail.com; 3 Kerecis^®^, 101 Reykjavik, Iceland; sm@kerecis.com (S.M.); hilmarkj@gmail.com (H.K.)

**Keywords:** swine, burn injury, skin regeneration, contraction, temporary covering, allograft, autologous graft, dermal substitute

## Abstract

The extent and depth of burn injury may mandate temporary use of cadaver skin (allograft) to protect the wound and allow the formation of granulation tissue while split-thickness skin grafts (STSGs) are serially harvested from the same donor areas. However, allografts are not always available and have a high cost, hence the interest in identifying more economical, readily available products that serve the same function. This study evaluated intact fish skin graft (IFSG) as a temporary cover to prepare the wound bed for STSG application. Thirty-six full-thickness (FT) 5 × 5 cm burn wounds were created on the dorsum of six anesthetized Yorkshire pigs on day −1. To mimic the two-stage clinical situation, on day 0, wounds were excised down to a bleeding wound bed and a temporary cover (either IFSG or cadaver porcine skin) was applied; then, on day 7, wounds were debrided to a viable wound bed prior to the application of autologous 1.5:1 meshed STSG (mSTSG). Rechecks were performed on days 14, 21, 28, 45, and 60 with digital images, non-invasive measurements, and punch biopsies. The IFSG created a granulated wound bed receptive to the application of an mSTSG. FT burn wounds treated with an IFSG had similar outcome measures, including contraction rates, trans-epidermal water loss (TEWL) measurements, hydration, and blood perfusion levels, compared to cadaver skin-treated burn wounds. Pathology scoring indicated significant differences between the allograft- and IFSG-treated wounds on day 7, with the IFSG having increased angiogenesis, granulation tissue formation, and immune cells. Pathology scoring indicated no significant differences once mSTSGs were applied to wounds. The IFSG performed as well as cadaver skin as a temporary cover and was not inferior to the standard of care, suggesting the potential to transition IFSGs into clinical use for burns.

## 1. Introduction

Data collected by the American Burn Association indicate that approximately 500,000 burn injuries occur in the United States every year, with roughly 40,000 hospitalizations and 3400 deaths occurring annually as a result [1]. Wound coverage via autograft following wound excision is the current gold standard of treatment for both deep partial-thickness (DPT) and full-thickness (FT) burns. Large total body surface area (TBSA) burns often require grafting and are hampered by issues including no donor site, inadequate amounts of donor skin available for harvesting, and donor site morbidity [2]. This gives rise to the need for off-the-shelf products to temporize the wound while the donor sites can heal for additional harvesting and skin grafting.

Biologic scaffolds include cryopreserved allografts (CPAs) harvested from human donors and xenografts sourced from mammalian extracellular matrices of non-human species, a common example of which is porcine xenograft. There are advantages and disadvantages for both CPAs and xenografts, with studies finding CPAs particularly useful due to their biocompatibility. However, the limited availability of CPAs brings to attention the need for a readily available off-the-shelf product that can provide temporary coverage for surgically debrided burns in the event that an autograft cannot yet be harvested. Once the necrotic tissue has been debrided, temporary coverage is needed in order to maintain moisture, prevent infection, and regulate temperature. A possible solution can be found with intact fish skin grafts (IFSGs) sourced from North Atlantic cod (*Gadus morhua*). IFSGs pose a wide variety of benefits, such as minimal processing requirements that allow the product to reserve its structure and lipid composition prior to usage. The minimal processing is possible due to the fact there is no known risk of transmitting prion or viral diseases from North Atlantic cod to humans, which serves as a possible advantage over CPAs [3]. Additionally, the structure of IFSGs compares more closely with human skin in comparison with other anti-viral processed skin substitutes, and naturally contains healthy polyunsaturated fatty acids [3]. An IFSG is a lyophilized, terminally sterilized, biological tissue which can be easily rehydrated, has a three-year shelf life at room temperature, and has been proven to be more cost-effective than standard-of-care treatments for chronic wounds [4,5]. IFSGs are available in individual pieces in sizes up to 300 sq cm, and, when meshed 2:1, the IFSG can be expanded to cover an area up to 540 sq cm. IFSGs have proven successful for a variety of indications, including burn wounds, acute trauma wounds, and chronic wounds such as diabetic foot ulcers, and are in further development for a variety of other conditions [6,7,8,9,10,11,12,13].

The purpose of this study was to evaluate IFSGs as a temporary cover to prepare the wound bed for STSG application in an established preclinical swine burn wound model. It was hypothesized that the IFSG would enhance granulation tissue formation that promote successful autografting, and the wounds will heal in a similar manner to allograft-treated wounds. This research is designed to evaluate preclinical benefits and risks for IFSGs as clinicians begin using IFSGs in human burn treatments.

## 2. Materials and Methods

### 2.1. Animals

This research was conducted in compliance with the Animal Welfare Act, the implementing Animal Welfare regulations, and the principles of the Guide for the Care and Use of Laboratory Animals. The Institutional Animal Care and Use Committee approved all research conducted in this study (A-16-021-TS5 approved 5 May 2017). AAALAC International fully accredited the research facility. Female Yorkshire swine (Midwest Research Swine, Gibbon, MN, USA) were utilized in this study (N = 6; 51.8 ± 3.3 kg). Prior to experimentation, the swine were acclimated to the facilities with full access to food and water for a minimum of seven days. 

This animal study was performed in 2017 as part of a Military Research Burn Program (MBRP) grant with different timelines and treatments for deep partial-thickness burns [14] and full-thickness burns (this manuscript). After the studies were completed, the data were compiled into two separate manuscripts. The intent of the project was never to compare the DPT burns to the FT burns, but only to evaluate the different treatments and how they were applied for each type of burn.

### 2.2. Allograft Preparation

Tissue was obtained via tissue sharing from a euthanized Yorkshire pig. Hair was removed from the area to be used for harvesting. Skin was cleaned with povidone-iodine solution. A dermatome was used to harvest partial-thickness skin from the pig’s back (ventral and dorsal trunk, left and right upper arm, and left and right thigh are all potential donor sites). Allografts were soaked in sterile saline with penicillin/streptomycin at 4 °C until further processing under a laminar flow hood. Under the laminar flow hood, allografts were soaked in 0.1% benzalkonium bromide for 15 min to sterilize, and were then washed for 10 min, 3 times, in normal saline with penicillin/streptomycin. For non-viable preservation, allografts were stored in 85% glycerol in the long term at 4 °C. Prior to use, allografts were brought to room temperature and rinsed with sterile saline for 30–60 min to remove any preservation residue [15,16].

### 2.3. Anesthesia

**Figure 1:**
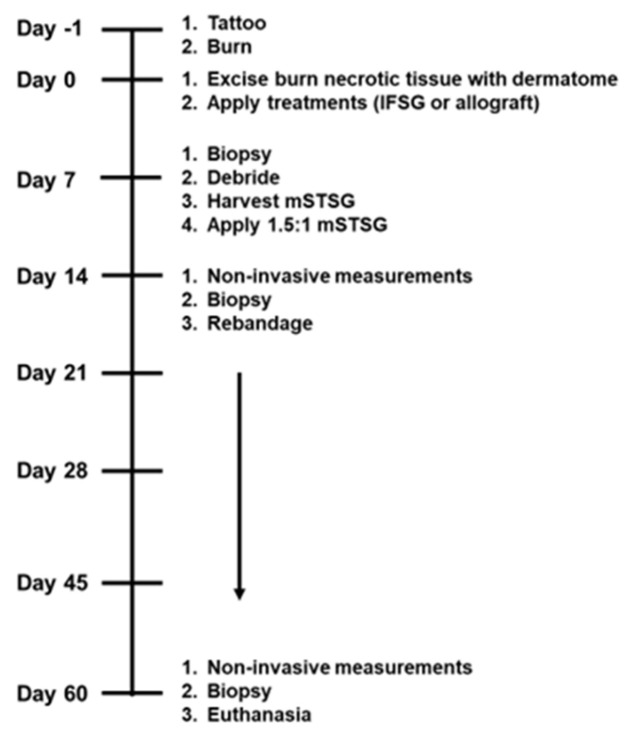
Preclinical experimental design. The 62-day swine experiment with aligned tasks is shown and was accomplished while under anesthesia at each time point. Non-invasive measurements include hydration, trans-epidermal water loss (TEWL), and imaging with a digital camera and laser speckle device. IFSG = fish skin graft; mSTSG = meshed split-thickness skin graft.

All procedures were conducted on anesthetized animals throughout the duration of the 62-day study, as shown in Figure 1. Additionally, on days 3, 10, and 35, digital imaging and dressing change occurred while under anesthesia. All animals were fasted the night prior to anesthesia and premedicated with glycopyrrolate (0.01 mg/kg) or atropine (0.2 mg/kg) via intramuscular injection (IM) in the neck region as needed in order to decrease saliva secretion and prevent vagally mediated bradycardia during the operation. Tiletamine-zolazepam (Telazol, 4–6 mg/kg, IM) or ketamine (10–25 mg/kg, IM) were used to induce the animals, and a facemask with 3–5% isoflurane in oxygen was used for the initial administration of anesthesia. Following intubation via an endotracheal tube, the pigs were put on an automatic ventilator with a peak pressure of 20 cm H_2_O and a respiration rate of 8–20 breaths per minute. The initial tidal volume was 8–12 mL/kg and the settings on the ventilator were adjusted to achieve an end tidal PCO_2_ of 40 ± 5 mmHg; 1% to 3% isoflurane in oxygen was used to maintain anesthesia. The administration of analgesia took place preemptively before wounding on day −1 and as needed with sustained-release buprenorphine (0.1–0.24 mg/kg) or hydromorphone (0.2–0.4 mg/kg) administered subcutaneously (SQ) in the lateral neck, dorsal lumbar, or caudal hamstring. Complete blood counts (CBC) were collected routinely throughout the study by harvesting blood from the external jugular via venipuncture. The CBC results from all 6 animals were previously reported in the DPT manuscript [14] and, to avoid duplication, are not presented in this manuscript.

### 2.4. Porcine Burn Wounds

The hair of each animal on the dorsum was chemically depilated using Nair™ (Church & Dwight, Ewing, NJ, USA) and was subsequently rinsed with sterile water. The perimeter of each wound was permanently marked with a square using a tattoo machine to create points of reference for burn creation, imaging, and re-epithelization and contraction calculations. Tattoos designating the wound number for each wound were tattooed on all animals. Ten square wounds measuring 25 cm^2^ were created on the dorsum of the anesthetized pigs in two evenly spaced rows located 2–3 cm from the spine, with 4 cm separating the wounds from one another. While under anesthesia, a thermo-coupled brass burn device heated to 100 °C and a force meter delivering constant pressure (~0.4 kg/cm^2^) were utilized to create six FT burns and four DPT burns on the dorsum of the pigs. Overall, 36 FT burns and 24 DPT burns were created amongst the 6 animals. All animals were also left with two control regions of unburned skin. This manuscript describes the outcomes of treating FT burns with IFSGs (Kerecis^®^ Omega 3 Burn, Kerecis^®^, Reykjavik, Iceland) or allografts as a temporary coverage followed by autografting. The results of the DPT burns were reported in the previous manuscript [14]. A Nikon digital camera and a Silhouette™ (Aranz Medical, Christchurch, New Zealand) hand-held 3D laser image capture device were used for imaging purposes for the duration of this study. Non-adherent sterile gauze (Telfa, Kendall, Mansfield, MA, USA) and an antiseptic occlusive dressing (Ioban™, 3M, St. Paul, MN, USA) were applied after the creation of the burns.

### 2.5. Surgical Debridement and Temporary Coverage Treatment

Wound dressings were removed from anesthetized animals on day 0 or one day post burn. The necrotic burn eschar was tangentially debrided down to a bleeding wound bed with a pneumatic dermatome (Zimmer, Warsaw, IN, USA). Serial passes of the dermatome set at 0.030″ were made until bleeding was observed. Then, an additional pass with the dermatome set to 0.010″ or a curette was utilized to remove any remaining necrotic burn tissue in order to have a completely viable ~25 cm^2^ wound bed. Four FT wounds on each animal were treated with IFSGs and two with porcine allografts for a total of 24 IFSG-treated and 12 allograft-treated wounds over 6 animals. Sterile saline was used to rehydrate and increase the pliability of the products prior to application. The products were stapled to the wounds and covered with sterile gauze and a bolster with antimicrobial Telfa (Kendall, Mansfield, MA, USA). To prevent infection and mechanical shearing, the gauze was placed between the bolsters and fixed with Elasticon (Johnson and Johnson, New Brunswick, NJ, USA) and a fabric vest jacket (DeRoyal, Powell, TN, USA) to ensure the IFSG would not move. 

### 2.6. Autograft Harvesting and Application

Wound dressings were removed from anesthetized animals on day 7. Wounds were briefly debrided via a combination of wet debridement and the use of a curette. Prior to any grafting, a biopsy of each wound bed was harvested. STSGs were harvested from the hindlimbs at 0.010–0.012″ with the pneumatic dermatome and meshed at 1.5:1 or 3:1 with a Zimmer mesher. On each animal, two IFSG-treated wounds received a 1.5:1 meshed STSG (mSTSG), two IFSG-treated wounds received a 3:1 mSTSG + another piece of IFSG as a protective cover, and the allograft-treated wounds received a 1.5:1 mSTSG. Grafted wounds were covered with a bolster, dressings, and jacket as performed on day 0. Since the 3:1 mSTSG was only evaluated with an IFSG and not an allograft as a control, the data are presented as supplemental (Supplemental Figures S1 and S2 and Table S1).

### 2.7. Wound Care

Following dressing removal, diluted 4% chlorhexidine gluconate (Hibeclens^®^, Molnlycke Health Care, Norcross, GA, USA) and sterile water were used to rinse the backs of the animals, which were subsequently dried using sterile gauze. A routine wound-cleaning protocol using wet debridement, sterile gauze, and sterile water was conducted on designated days to ensure wound cleanliness and remove peripheral exudate.

### 2.8. Contraction Calculations

Digital images were captured with a Silhouette™ device (Aranz Medical, Christchurch, New Zealand) throughout the experiment. The SilhouetteConnect^TM^ software (version: 3.22.0, Aranz Medical, Christchurch, New Zealand) allows for the tracing of multiple areas of interest to acquire the wound area. The tattoos surrounding the wounds were traced at all time points to determine the contraction of each wound. To account for the increase in surface area that occurred via the growth of the animal during the 62-day study, each wounds’ calculated area was normalized to the growth controls on that particular animal and is represented as ‘*Normalized Tattoo (Dx)*’, with D meaning day and x as the time point. To calculate contraction rates, we used the following equation to generate the percent original wound size, with 100% representing the original wound size.

$$\%\;\mathrm{Original}\;\mathrm{Wound}\;\mathrm{Size}=(1-\frac{\left[Tattoo\;\left(D0\right)-Normalized\;Tattoo\;\left(Dx\right)\right]}{Tattoo\;\left(D0\right)})*100$$

### 2.9. Skin Function Measurements

Skin barrier properties were measured using a DermaLab Combo trans-epidermal water loss (TEWL) and hydration device (Cortex Technology, Hadsund, Denmark). The density gradient of water loss from the skin due to evaporation is measured using TEWL and shown as grams per hour per square meter. The hydration probe assesses the stratum corneum’s capacity for water binding. Three measurements were evaluated for TEWL (cranial, middle, and caudal) and five measurements for hydration (cranial, middle, caudal, medial, and lateral). Measurements for all wounds at each time point were then averaged. Three measurements were taken for TEWL as opposed to five because each TEWL reading took up to 2 min as compared to less than 5 s per reading for hydration. 

### 2.10. Laser Speckle

Perfusion of the wounds was acquired by placing a high-resolution laser speckle imager (LSI; Moore FLPI Instruments Inc., Wilmington, DE, USA) approximately 10–20 cm from the injured skin. Acquisition settings for the images were as follows: gain: 200; exposure time: 4 ms; time constant: 1.0 s; filter: 250 frames; sample interval: 20 s; image resolution: 760 × 568. To perform image analysis, all frames were merged together into one image and three areas of interest were drawn: one area of wound flux, one area of normal skin perfusion (background flux) to the left of the wound, and one area of normal skin perfusion to the right of the wound. The fold change in Median Wound Flux/Median Background Flux was calculated for each wound.

### 2.11. Histology and Pathological Analysis

Biopsy punches measuring 8 mm in diameter were acquired prior to application of the mSTSGs on day 7. Additional biopsy punches were harvested following non-invasive measurements on days 14, 21, 28, and 45. At the completion of the study on day 60, a strip biopsy covering the entirety of the wound bed was harvested. Samples were placed in 10% neutral buffered formalin for a minimum of 48 h before paraffin embedding. Blocks of tissue were cut and placed on a Superfrost^®^ Plus slide once all samples were paraffin-embedded. Xylene was used to clear the slides and a mixture of 100% ethanol, 95% ethanol, 70% ethanol, and DI water was then used to rehydrate the slides. Standard hematoxylin and eosin (H&E), Masson’s trichrome staining (MTS, Sigma Aldrich^®^, St. Louis, MO, USA), and α-smooth muscle actin (α-SMA) staining were performed as previously described [14,17]. An Axio Scan.Z1 (Zeiss, Oberkochen, Germany) at 20× was used to scan the slides and were exported to JPEGs. Slides were blindly scored by a board-certified veterinary pathologist in accordance with the scoring system shown in Table 1 and Table 2.

### 2.12. Statistical Analysis

The statistical analysis for the pathology scoring is separated into 2 phases: before grafting (day 7) and after grafting (D14–60). Since the day 7 assessment was of the wound bed after the application of the temporary coverage products but before any grafting, the sample size was 12 for allografts and 24 for IFSGs. We observed 2 graft failures for the IFSG treated with the 1.5:1 mSTSG on day 14; hence, all analysis from D14–D60 was with the remaining 10 wounds. For pathology scoring analysis comparing two groups, the nonparametric Mann–Whitney test was used to test for differences in mean rank scores. Two-way repeated-measures ANOVA with Tukey or Sidak’s post hoc test for multiple comparisons were used to determine if statistically significant differences were observed for contraction, TEWL, hydration, and LSI via GraphPad Prism 8.2.1 (GraphPad Software Inc., La Jolla, CA, USA). The TEWL, hydration, and LSI data were not normally distributed; therefore, those datasets required log transformation to meet normal distribution. These datasets were reported as the mean ± confidence interval (CI). We also observed 2 graft failures for the IFSG treated with the 3:1 mSTSG on day 14; hence, all analysis with the 3:1 group from D14–D60 was with the remaining 10 wounds. For pathology scoring analysis comparing all three groups, the Kruskal–Wallis test with Dunn’s multiple comparison test was used to test for differences in mean rank scores stratified by time. Statistical significance was found when *p* < 0.05 and is indicated in the figures.

## 3. Results

### 3.1. Porcine Burn Wound Model

**Figure 2:**
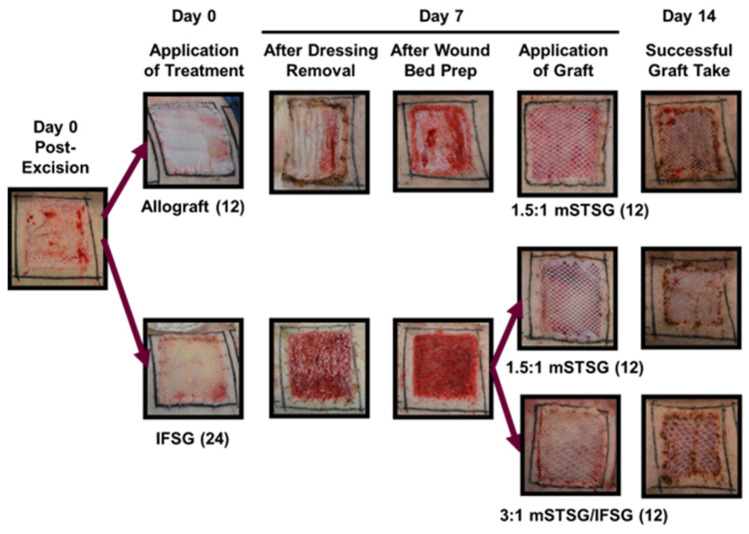
Representative digital images of the treatment groups. The schematic indicates the procedures that occurred on days 0, 7, and 14 with associated images captured during this study. All wounds were photographed throughout the study. IFSG = intact fish skin graft; mSTSG = meshed split-thickness skin graft. Number in parenthesis represents the number of wounds receiving that treatment on the days indicated.

Figure 1 illustrates the timeline followed over the course of the 62-day experiment. Figure 2 shows how the treatments appeared on the wounds after debridement on day 0 up until day 14. The animals were treated in pairs, and on the first two animals, all bolsters were dampened with saline. On these two animals, seroma formation was observed in two of the IFSG-treated wounds. We hypothesized that the additional saline may have been a contributing factor, and for the remaining four animals, dry bolsters were used for the IFSG only. The day-7 images show a more granulated wound bed after wound bed prep for the IFSG compared to the allograft. To fully expand the 1:1.5 meshed IFSG product, additional staples around the periphery of wound were needed. With these two extra measures in place, seromas were observed on day 7 in 2/16 of the IFSG-treated wounds on the remaining four animals. 

### 3.2. Histological Analysis of Wound Bed after 7 Days of Temporary Coverage of IFSG or Allograft

A board-certified veterinary pathologist was blinded to all groups to remove bias and the pathologist scored H&E sections using the rubric in Table 1. The scoring results are shown for each parameter on day 7 prior to grafting. At this point, 12 wounds had received an allograft and 24 wounds had received an IFSG for 7 days as a temporary covering. Statistically significant differences were observed for angiogenesis, granulation tissue/fibroplasia, and all immune cells with increased levels in the IFSG-treated wounds compared to the allografts.

### 3.3. FSG Stimulated a Granulated Wound Bed

**Figure 3:**
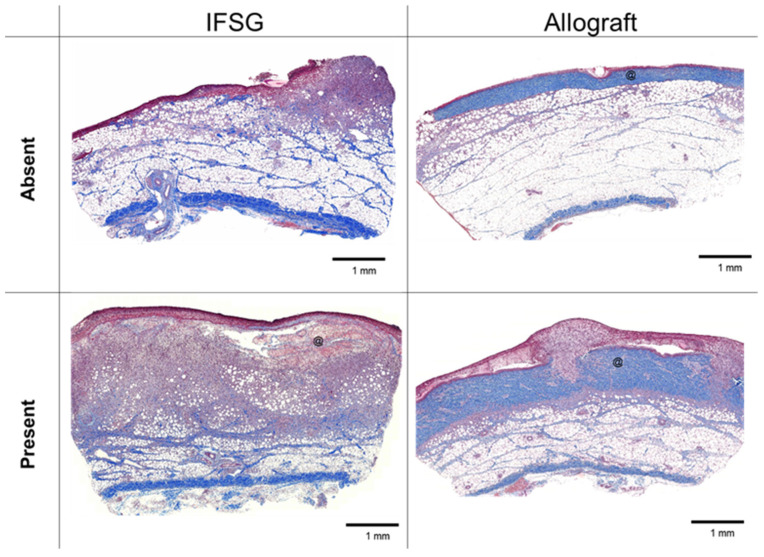
Mason’s trichrome histology at day 7 with granulation tissue formation. Representative images of biopsy punches harvested at the day-7 time point prior to skin graft application that align with the pathologist scoring of granulation tissue shown in Table 2. Black scale bars = 1 mm. @ = residual pieces of treatment in wound bed. IFSG = intact fish skin graft.

**Figure 4:**
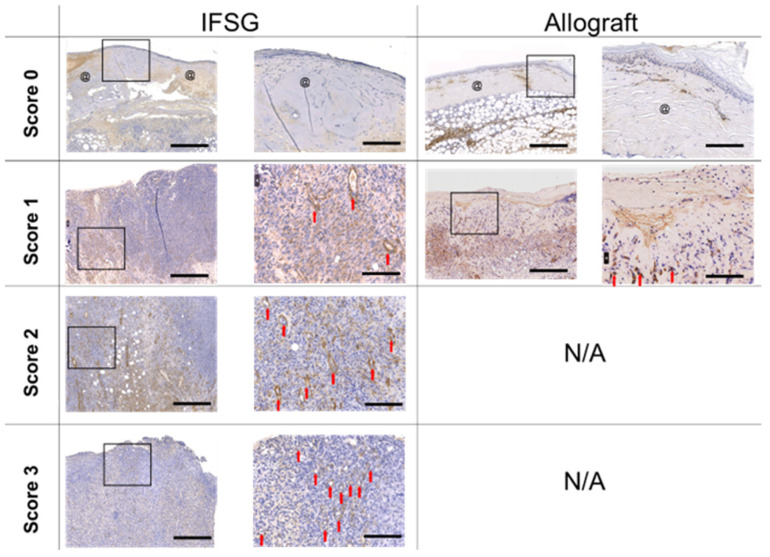
Day 7 alpha-smooth muscle actin staining. Representative images of slides stained with α-SMA that consist of wound bed samples collected prior to skin graft application on day 7. Sections are shown that align with the pathologist scoring listed in Table 2. No allograft-treated wounds scored a 2 or 3 and, hence, N/A is indicative of no wounds with those scores. Myofibroblasts and new blood vessels can be identified in the images by the positive brown staining. The left image of the biopsy punch has scale bars = 500 µm. The image on the right is the magnified black box region from the left image with scale bars = 125 µm. Residual pieces of product = @; new blood vessels = red arrow. IFSG = intact fish skin graft.

Figure 3 shows representative Masson’s trichrome-stained images on day 7 for both IFSG- and allograft-treated wounds. Examples are shown that align with the pathologist scoring of either absent or present. Eighty three percent (20/24) of the IFSG-treated wounds had observable amounts of granulation tissue as opposed to only 16.7% (2/10) for the allograft-treated wounds. To visualize newly formed blood vessels, sections were stained with alpha-smooth muscle actin (α-SMA). Figure 4 shows sections stained with α-SMA with IFSGs spanning the entire range of scores used by the pathologist, while the allografts resulted in minimal new blood vessel formation by day 7.

### 3.4. Wounds Treated with IFSG Showed No Differences in Contraction, Perfusion, TEWL, or Hydration Compared to Allograft after Grafting

**Figure 5:**
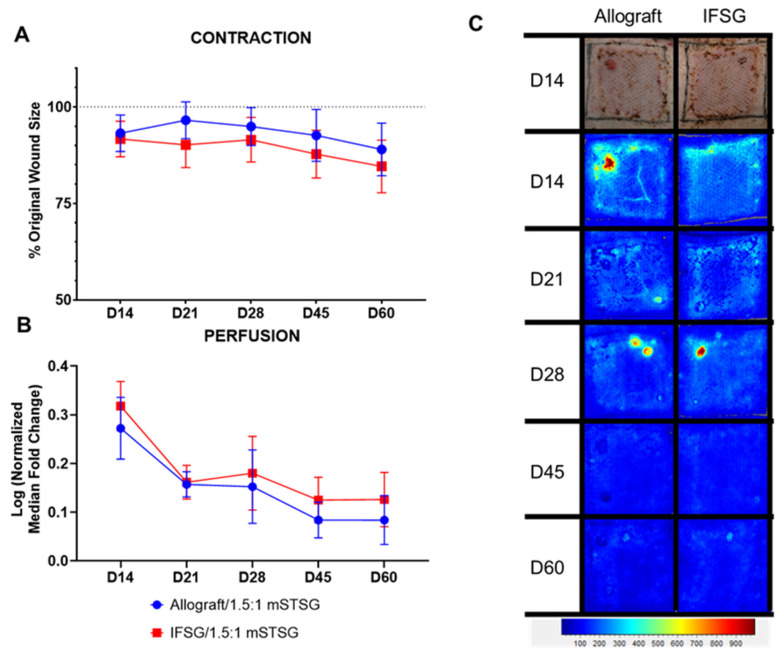
Contraction and laser speckle imaging. (**A**) Wound contraction was calculated by tracing the tattoos, compared to the initial wound size, and normalized to the growth of each animal. The wound size on day 0 is represented by the dotted line. If contraction occurred during healing, a decrease in the % original wound size would be observed. No significant differences comparing the groups at each time point were detected by 2-way repeated-measures ANOVA with Sidak’s post hoc test. (**B**) Laser speckle imaging was used to measure the perfusion in the wounds. A fold change was calculated by dividing the wound perfusion by the local background perfusion. The perfusion within the growth control regions was used for normalization for each time point. The normalized median fold change was log transformed in order to pass the Shapiro–Wilk normality test. No significant differences comparing the groups at each time point were detected using two-way repeated-measures ANOVA with Sidak’s post hoc test. The median with 95% confidence intervals is represented in the figure; N = 10 (IFSG); 12 (allograft). (**C**) One wound is depicted for each group throughout the study. A day-14 digital image is shown with the longitudinal LSI images. At the bottom is the heatmap scale that defines blue as low perfusion and red as high perfusion. Normal uninjured skins’ baseline perfusion is blue. By the day 60 end-point, the wound edges are no longer obvious, suggesting a return to normal perfusion levels. IFSG = intact fish skin graft.

**Figure 6:**
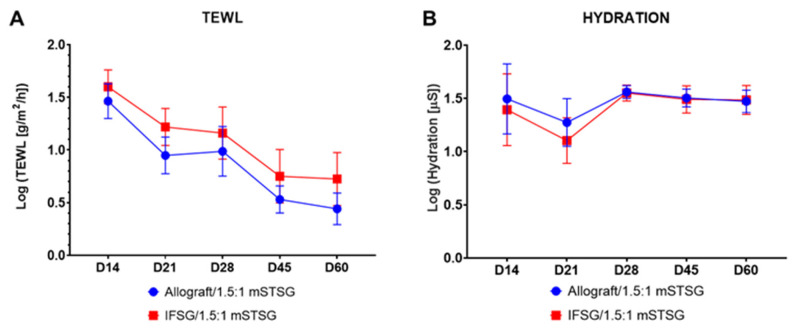
Barrier function of healing burn wounds. (**A**) The skin barrier function was measured by TEWL for all wounds. The mean TEWL measurement was obtained by averaging 3 spots from each wound per time point. The mean was log transformed in order to pass the Shapiro–Wilk normality test. No significant differences comparing the groups at each time point were detected by 2-way repeated-measures ANOVA with Sidak’s post hoc test. (**B**) Hydration measures the water content of the wounds. Hydration values of 5 spots within each wound were averaged. The mean was log transformed in order to pass the Shapiro–Wilk normality test. No significant differences comparing the groups at each time point were detected by 2-way repeated-measures ANOVA with Sidak’s post hoc test. Results are shown as mean with 95% confidence intervals; N = 10 (IFSG); 12 (allograft). IFSG = intact fish skin graft.

**Figure 7:**
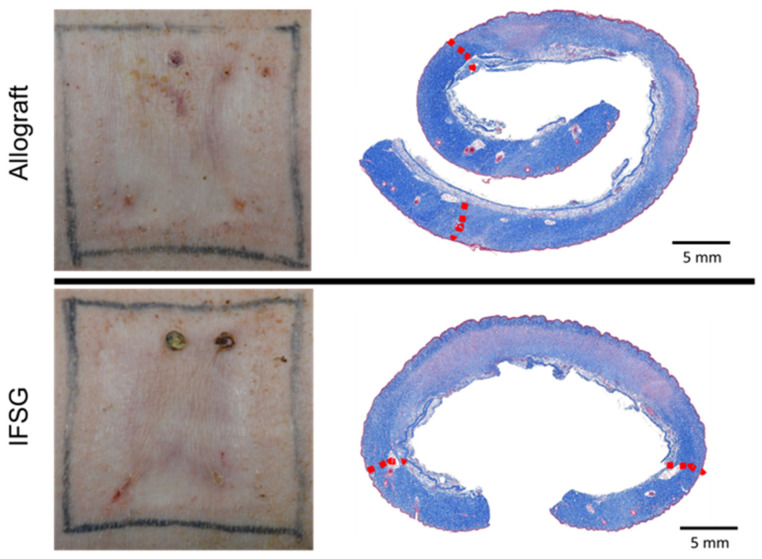
Representative digital images at end of the study (day 60). Representative digital images of each treatment group are shown. No obvious meshed pattern scarring is present at the day-60 final time point. Small wounds/scabs are present as a result of biopsy punch sites. Corresponding Masson’s trichrome staining images on day 60 indicating similar overall wound healing in the IFSG-treated wounds compared to allografts. Red dotted lines indicate approximate wound edges. IFSG = intact fish skin graft.

Wound closure was assessed by determining the contraction rates and reported as the percent of the original wound size. Figure 5A shows that the wounds contracted in a similar manner, with no significant differences detected between the two treatment groups. Figure 5B shows the quantitative evaluation of blood perfusion as measured by laser speckle, with the accompanying illustrative images in Figure 5C. The basal amount of blood flow is represented by the light-blue region and scales up to red with increasing perfusion. Since all wounds were grafted with a 1.5:1 mSTSG, only the interstitial spaces needed to fill in and re-epithelialize. The first assessment was 7 days post grafting (on day 14), hence the low perfusion levels observed in the wounds. No significant differences in perfusion levels were detected between the two treatment groups. The wounds were assessed for function over time by measuring both TEWL and hydration (Figure 6). No significant differences in TEWL or hydration levels were detected between the two treatment groups. Figure 7 shows representative images of how similar the wounds appeared on day 60 from a digital and histological perspective.

### 3.5. Histological Analysis after Grafting

A board-certified veterinary pathologist was blinded to the groups to remove bias and scored H&E sections using the same rubric. Table 2 shows the scoring for each parameter from day 14 through to Day 60. No significant differences were detected between the two treatment groups for any parameter at any time point.

**Table 2:** Histology assessing wound bed after grafting. A veterinary pathologist blinded to the groups scored the sections from day 14 through day 60 for the stated parameters. The 2 groups were compared by performing a nonparametric Mann–Whitney test to compare ranks for statistical significance. The *p* values are indicted for each parameter comparing the allograft wounds to the IFSG-treated wounds. No statistical differences were found for any parameter at any time point. IFSG = intact fish skin graft; IC = inflammatory cell.

Pathologist Scoring Rubric	Allograft/1.5:1 mSTSG (N = 12)					IFSG/1.5:1 mSTSG (N = 10)				
**Epidermal Status**	**D14**	**D21**	**D28**	**D45**	**D60**	**D14**	**D21**	**D28**	**D45**	**D60**
0 = No epidermis	0	0	0	0	0	0	0	0	0	0
1 = Partial epidermis	0	0	0	0	0	1	0	0	0	0
2 = Regenerating or hyperplastic with 100% coverage	10	4	0	1	0	6	3	1	0	1
3 = Normal epidermis across entire wound bed	2	8	12	11	12	3	7	9	10	9
*p* value	0.801	>0.999	0.455	>0.999	0.455					
**Fibroplasia**	**D14**	**D21**	**D28**	**D45**	**D60**	**D14**	**D21**	**D28**	**D45**	**D60**
0 = None	0	1	0	0	0	0	0	0	0	0
1 = Minimal	0	0	1	0	0	0	0	0	0	0
2 = Mild	6	5	7	9	5	2	2	5	7	3
3 = Moderate	6	6	4	3	7	8	7	4	3	6
4 = Marked	0	0	0	0	0	0	1	1	0	1
*p* value	0.204	0.126	0.348	>0.999	0.526					
**Foreign Material**	**D14**	**D21**	**D28**	**D45**	**D60**	**D14**	**D21**	**D28**	**D45**	**D60**
0 = Absent	7	12	12	12	12	9	10	10	10	10
1 = Present	5	0	0	0	0	1	0	0	0	0
*p* value	0.162	>0.999	>0.999	>0.999	>0.999					
**Angiogenesis**	**D14**	**D21**	**D28**	**D45**	**D60**	**D14**	**D21**	**D28**	**D45**	**D60**
0 = None	8	9	10	12	12	4	7	8	9	10
1 = ≤ 50 Vessels	4	3	2	0	0	6	3	2	1	0
*p* value	0.391	>0.999	>0.999	0.455	>0.999					
**Hemorrhage**	**D14**	**D21**	**D28**	**D45**	**D60**	**D14**	**D21**	**D28**	**D45**	**D60**
0 = Absent	8	8	9	10	8	7	5	8	7	6
1 = Present	4	4	3	2	4	3	5	2	3	4
*p* value	>0.999	0.666	>0.999	0.624	>0.999					
**Hemorrhage Severity**	**D14**	**D21**	**D28**	**D45**	**D60**	**D14**	**D21**	**D28**	**D45**	**D60**
0 = Absent	8	8	9	10	8	7	5	8	7	6
1 = Mild	4	4	3	2	3	3	5	2	3	4
2 = Moderate	0	0	0	0	1	0	0	0	0	0
*p* value	>0.999	0.666	>0.999	0.624	>0.999					
**Neutrophils**	**D14**	**D21**	**D28**	**D45**	**D60**	**D14**	**D21**	**D28**	**D45**	**D60**
0 = None	10	11	12	12	12	7	10	9	10	10
1 = Minimal number of ICs	2	1	0	0	0	3	0	1	0	0
2 = Mild number of ICs	0	0	0	0	0	0	0	0	0	0
*p* value	0.624	>0.999	0.455	>0.999	>0.999					
**Eosinophils**	**D14**	**D21**	**D28**	**D45**	**D60**	**D14**	**D21**	**D28**	**D45**	**D60**
0 = None	8	11	10	12	12	6	8	10	10	9
1 = Minimal number of ICs	4	1	2	0	0	2	2	0	0	1
2 = Mild number of ICs	0	0	0	0	0	2	0	0	0	0
*p* value	0.554	0.571	0.481	>0.999	0.455					
**Lymphocytes**	**D14**	**D21**	**D28**	**D45**	**D60**	**D14**	**D21**	**D28**	**D45**	**D60**
0 = None	7	8	7	11	8	4	3	8	8	5
1 = Minimal number of ICs	4	3	4	1	3	5	6	1	2	3
2 = Mild number of ICs	1	1	1	0	1	1	1	1	0	2
*p* value	0.481	0.148	0.448	0.571	0.513					
**Macrophages**	**D14**	**D21**	**D28**	**D45**	**D60**	**D14**	**D21**	**D28**	**D45**	**D60**
0 = None	7	10	8	9	7	5	9	8	8	5
1 = Minimal number of ICs	4	2	4	3	5	5	1	2	2	4
2 = Mild number of ICs	1	0	0	0	0	0	0	0	0	1
*p* value	>0.999	>0.999	0.646	>0.999	0.726					

## 4. Discussion

The future multi-domain battlefield is expecting delayed evacuation times of days to up to a week, resulting in a prolonged field care (PFC) scenario. In the most recent military conflicts, burns accounted for 5–10% of injuries [18,19], which, even for small burns, require more resources for definitive care [20,21]. It is anticipated that future conflicts may have higher incidences of burn injuries. Given this possibility, the identification of better point-of-injury products for burn care is paramount. Current doctrine according to the Joint Trauma System Clinical Practice Guidelines for Burn Care in a PFC environment states that excision and grafting of the burn wound should occur at the US Army Institute of Surgical Research [22]. Although the idea of non-surgical debridement at lower echelons of care is currently being explored, it would require the use of a product that can act as a temporary covering, such as an IFSG (as well as specialized multidisciplinary training and procedures).

In this study, full-thickness burns were created on six anesthetized swine. The burns were surgically debrided, treated with two temporary coverings (IFSG and allograft), and evaluated for long-term wound healing. Both treatments were successful in providing temporary coverage, while the IFSG resulted in a more granulated wound bed. The IFSG product was easy to apply to wounds and not conducive to infection. Specifically, the path scoring for pustules on day 7 was 2/12 for cadavers and 0/20 for IFSGs, and no infection was observed macroscopically. There were issues with primary/secondary dressings used for the IFSG. Further optimization was needed to ensure that the product was not removed at dressing changes. This study was not powered to evaluate graft loss comparing the IFSG to the allograft, and a larger study may be warranted. Two other issues occurred during the experiment with the IFSG-treated wounds: seroma formation and graft failures (although not statistically significant). We observed seromas in four full-thickness burn wounds treated with the IFSG. The IFSG was meshed at 1.5:1, and it is possible that we did not expand the material as much as possible to allow the exudate to pass through. We observed a hyper-granulated wound bed, and the seroma formation could have been from the accumulation of fluid leaking between the wound bed and IFSG. Consequently, since this was the first time we had encountered such a hyper-granulated wound bed, our attempts at debridement prior to skin grafting may have been inadequate and resulted in the subsequent graft failure noted in four wounds (two for the 1.5:1 and two for the 3:1 mSTSG) initially treated with the IFSG. Overall, both treatments provided temporary coverage that (1) prevented infection and (2) induced the formation of a granulated wound bed for successful grafting. In addition, the FT burn wounds treated with IFSGs had similar outcome measures (contraction, TEWL, conductance, blood perfusion) compared to the allograft-treated wounds. Interestingly, the 3:1 mSTSG + IFSG resulted in similar healing compared to the wounds treated with the 1.5:1 mSTSG, meaning that, ultimately, less autograft (~25%) was necessary to result in similar healing without any meshed pattern being exhibited. This is a desired outcome as this work is translated into human clinical use.

The limitations of this study are that this was evaluated in an animal model and may not translate to humans. The wounds were small (5 × 5 cm^2^); therefore, we cannot predict how these products may work on a large TBSA burn. The small samples size (six animals with 12 wounds total for each treatment) is a concern. No untreated wounds (negative control) were included on these animals, no comparison to any synthetic dressing (e.g., Biobrane, Mepilex, Suprathel, etc.) was made, and no composite histology score was devised for this study. The application of the secondary dressing had not been fully optimized and we had to make changes after the first set of animals. The IFSG was applied twice on the widely 3:1 meshed grafted wounds—there was no control group with allograft + 3:1 + allograft—hence the reason to only show the data as supplemental (Supplemental Figures S1 and S2, and Table S1). We could have performed additional final wound assessments for scarring via a POSAS, erythema/melanin measurements, and/or picrosirius staining. In addition, a recommendation for any future work would be to extend the length of the study and utilize Red Duroc pigs as they are the accepted strain for evaluating scarring [23,24]. Although, considering that we found no differences in our current assessments, there is no reason to believe that these additional measurements would have been different.

In this study, an IFSG demonstrated its ability to act as a temporary covering for full-thickness burn wounds after proper surgical debridement. It promoted increased granulation tissue by day 7 without infection and allowed for successful graft take. During manufacturing, the IFSG covering endures minimal processing, is easy to rehydrate and apply to wounds, and is more culturally acceptable compared to other commercially available animal-derived products for burn wounds. From a military perspective, it has a potential to fill a capability gap as a temporary coverage for burn injuries in humans after surgical or non-surgical debridement. Future studies should include non-surgical debridement followed by placement of products like IFSGs.

## Figures and Tables

**Table 1:** Histology assessing wound bed before grafting. A veterinary pathologist blinded to the groups scored the sections on day 7 for the stated parameters. The 2 groups were compared by performing the nonparametric Mann–Whitney test to compare ranks for statistical significance. The *p* value was determined for each parameter comparing the allograft wounds to the IFSG-treated wounds, with ‘*’ notating when the value is less than 0.05 and therefore statistically significant. IFSG = intact fish skin graft; IC = inflammatory cell.

DAY 7 ANALYSIS		Allograft (N =12)		IFSG (N = 24)		*p* Value
Parameter	Pathologist Scoring Rubric	N	%	N	%	
**Angiogenesis**	0—None	6	50	3	12.5	<0.0001 *
	1—≤50 Vessels	6	50	3	12.5	
	2—51–100 Vessels	0	0	12	50	
	3—101–150 Vessels	0	0	6	25	
**Foreign** **Material**	0—Absent	6	50	5	20.8	0.1245
	1—Present	6	50	19	79.2	
**Hemorrhage**	0—Absent	6	50	4	16.7	0.0532
	1—Present	6	50	20	83.3	
**Hemorrhage Severity**	0—Absent	6	50	4	16.7	0.2493
	1—Mild	2	16.7	12	50	
	2—Moderate	4	33.3	7	29.2	
	3—Severe	0	0	1	4.2	
**Granulation Tissue**	0—Absent	10	83.3	4	16.7	0.0002 *
	1—Present	2	16.7	20	83.3	
**Fibroplasia**	0—None	1	8.3	0	0	0.0006 *
	1—Minimal	2	16.7	1	4.2	
	2—Mild	6	50	4	16.7	
	3—Moderate	3	25	11	45.7	
	4—Marked	0	0	7	29.2	
	5—Severe	0	0	1	4.2	
**Neutrophils**	0—None	4	33.3	0	0	<0.0001 *
	1—Minimal number of ICs	7	58.3	2	8.3	
	2—Mild number of ICs	0	0	13	54.2	
	3—Moderate number of ICs	0	0	2	8.3	
	4—Marked number of ICs	0	0	3	12.5	
	5—Severe number of ICs	1	8.4	4	16.7	
**Eosinophils**	0—None	7	58.3	1	4.2	<0.0001
	1—Minimal number of ICs	4	33.3	2	8.3	
	2—Mild number of ICs	1	8.3	12	50	
	3—Moderate number of ICs	0	0	9	37.5	
**Lymphocytes**	0—None	4	33.3	1	4.2	<0.0001 *
	1—Minimal number of ICs	5	41.7	0	0	
	2—Mild number of ICs	3	25	17	70.8	
	3—Moderate number of ICs	0	0	6	25	
**Macrophages**	0—None	6	50	1	4.2	0.0048 *
	1—Minimal number of ICs	5	41.7	16	66.7	
	2—Mild number of ICs	1	8.3	7	29.2	

## Data Availability

Due to the size of the raw files, datasets are available upon request.

## Supplementary Materials

The following supporting information can be downloaded at https://www.mdpi.com/article/10.3390/biomedicines12030680/s1: Figure S1: Digital and laser speckle images of IFSG/3:1 mSTSG-treated wounds; Figure S2: Box and whisker plot with 95% confidence intervals; Table S1: Histology assessing wound bed after grafting of IFSG + 3:1 mSTSG/IFSG group.

## References

[1] ABA (2016). Burn Incidence and Treatment in the United States: 2016 Fact Sheet.

[2] Stone R., Natesan S., Kowalczewski C.J., Mangum L.H., Clay N.E., Clohessy R.M., Carlsson A.H., Tassin D.H., Chan R.K., Rizzo J.A. (2018). Advancements in Regenerative Strategies through the Continuum of Burn Care. Front. Pharmacol..

[3] Magnusson S., Baldursson B.T., Kjartansson H., Rolfsson O., Sigurjonsson G.F. (2017). Regenerative and Antibacterial Properties of Acellular Fish Skin Grafts and Human Amnion/Chorion Membrane: Implications for Tissue Preservation in Combat Casualty Care. Mil. Med..

[4] Winters C., Kirsner R.S., Margolis D.J., Lantis J.C. (2020). Cost Effectiveness of Fish Skin Grafts versus Standard of Care on Wound Healing of Chronic Diabetic Foot Ulcers: A Retrospective Comparative Cohort Study. Wounds.

[5] Zehnder T., Blatti M. (2022). Faster than Projected Healing in Chronic Venous and Diabetic Foot Ulcers When Treated with Intact Fish Skin Grafts Compared to Expected Healing Times for Standard of Care: An Outcome-Based Model from a Swiss Hospital. Int. J. Low. Extrem. Wounds.

[6] Alam K., Jeffery S.L.A. (2019). Acellular Fish Skin Grafts for Management of Split Thickness Donor Sites and Partial Thickness Burns: A Case Series. Mil. Med..

[7] Kirsner R.S., Margolis D.J., Baldursson B.T., Petursdottir K., Davidsson O.B., Weir D., Lantis J.C. (2020). Fish skin grafts compared to human amnion/chorion membrane allografts: A double-blind, prospective, randomized clinical trial of acute wound healing. Wound Repair Regen..

[8] Lullove E.J., Liden B., Winters C., McEneaney P., Raphael A., Lantis J.C. (2021). A Multicenter, Blinded, Randomized Controlled Clinical Trial Evaluating the Effect of Omega-3-Rich Fish Skin in the Treatment of Chronic, Nonresponsive Diabetic Foot Ulcers. Wounds.

[9] Luze H., Nischwitz S.P., Smolle C., Zrim R., Kamolz L.P. (2022). The Use of Acellular Fish Skin Grafts in Burn Wound Management-A Systematic Review. Medicina.

[10] Michael S., Winters C., Khan M. (2019). Acellular Fish Skin Graft Use for Diabetic Lower Extremity Wound Healing: A Retrospective Study of 58 Ulcerations and a Literature Review. Wounds.

[11] Seth N., Chopra D., Lev-Tov H. (2022). Fish Skin Grafts with Omega-3 for Treatment of Chronic Wounds: Exploring the Role of Omega-3 Fatty Acids in Wound Healing and a Review of Clinical Healing Outcomes. Surg. Technol. Int..

[12] Woodrow T., Chant T., Chant H. (2019). Treatment of diabetic foot wounds with acellular fish skin graft rich in Omega-3: A prospective evaluation. J. Wound Care.

[13] Yang C.K., Polanco T.O., Lantis J.C. (2016). A Prospective, Postmarket, Compassionate Clinical Evaluation of a Novel Acellular Fish-skin Graft Which Contains Omega-3 Fatty Acids for the Closure of Hard-to-heal Lower Extremity Chronic Ulcers. Wounds.

[14] Stone R., Saathoff E.C., Larson D.A., Wall J.T., Wienandt N.A., Magnusson S., Kjartansson H., Natesan S., Christy R.J. (2021). Accelerated Wound Closure of Deep Partial Thickness Burns with Acellular Fish Skin Graft. Int. J. Mol. Sci..

[15] Kearney J.N. (2005). Guidelines on processing and clinical use of skin allografts. Clin. Dermatol..

[16] Chiu T., Burd A. (2005). “Xenograft” dressing in the treatment of burns. Clin. Dermatol..

[17] Stone R., Jockheck-Clark A.R., Natesan S., Rizzo J.A., Wienandt N.A., Scott L.L., Larson D.A., Wall J.T., Holik M.A., Shaffer L.J. (2020). Enzymatic Debridement of Porcine Burn Wounds via a Novel Protease, SN514. J. Burn Care Res..

[18] White C.E., Renz E.M. (2008). Advances in surgical care: Management of severe burn injury. Crit. Care Med..

[19] Chung K.K., Blackbourne L.H., Wolf S.E., White C.E., Renz E.M., Cancio L.C., Holcomb J.B., Barillo D.J. (2006). Evolution of burn resuscitation in operation Iraqi freedom. J. Burn Care Res..

[20] Wolf S.E., Kauvar D.S., Wade C.E., Cancio L.C., Renz E.P., Horvath E.E., White C.E., Park M.S., Wanek S., Albrecht M.A. (2006). Comparison between civilian burns and combat burns from Operation Iraqi Freedom and Operation Enduring Freedom. Ann. Surg..

[21] Owens B.D., Kragh J.F., Wenke J.C., Macaitis J., Wade C.E., Holcomb J.B. (2008). Combat wounds in operation Iraqi Freedom and operation Enduring Freedom. J. Trauma.

[22] Cancio L.C., Barillo D.J., Kearns R.D., Holmes J.H.t., Conlon K.M., Matherly A.F., Cairns B.A., Hickerson W.L., Palmieri T. (2017). Guidelines for Burn Care under Austere Conditions: Surgical and Nonsurgical Wound Management. J. Burn Care Res..

[23] Blackstone B.N., Kim J.Y., McFarland K.L., Sen C.K., Supp D.M., Bailey J.K., Powell H.M. (2017). Scar formation following excisional and burn injuries in a red Duroc pig model. Wound Repair Regen..

[24] Harunari N., Zhu K.Q., Armendariz R.T., Deubner H., Muangman P., Carrougher G.J., Isik F.F., Gibran N.S., Engrav L.H. (2006). Histology of the thick scar on the female, red Duroc pig: Final similarities to human hypertrophic scar. Burns.

