# Supplementary material for: Comparison of Intact Fish Skin Graft and Allograft as Temporary Coverage for Full-Thickness Burns: A Non-Inferiority Study
Source: Biomedicines. 2024 Mar 18;12(3):680. doi: 10.3390/biomedicines12030680 (PMC10967914; doi:10.3390/biomedicines12030680)
Supplement: Supplementary file 1 [file biomedicines-12-00680-s001.zip › Stone_FishSkin_Biomedicines_Supplemental.pdf]

biomedicines

MDPI

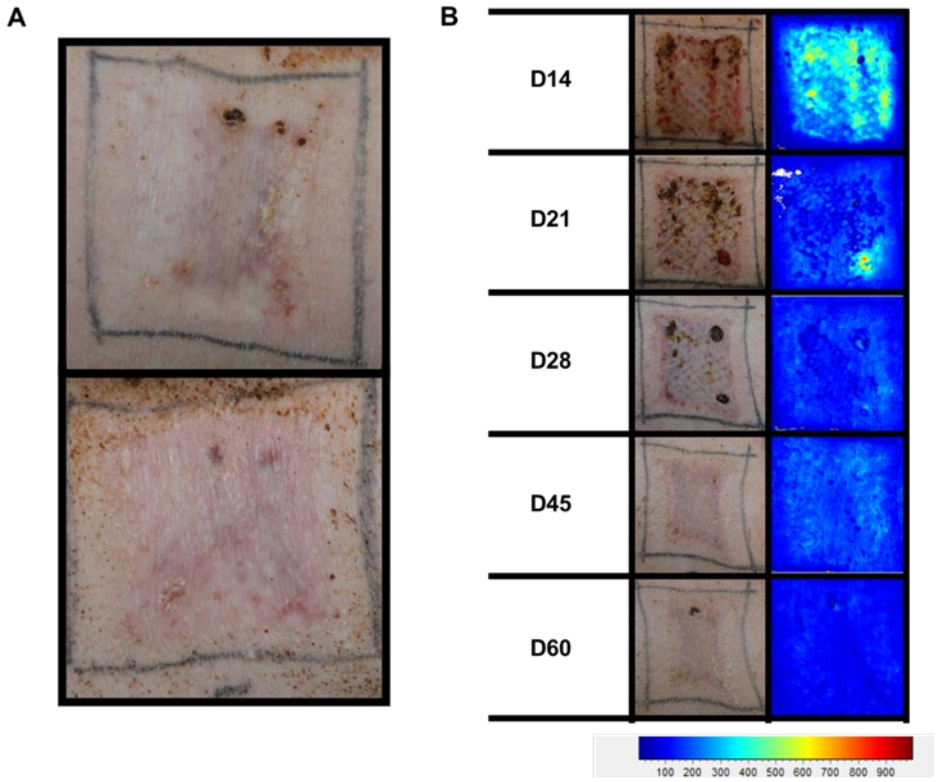

Supplemental Figure S1. Digital and Laser Speckle Images of FSG/3:1 mSTSG Treated Wounds. A) Representative digital images of the FSG/3:1 mSTSG group are shown. No obvious meshed pattern scarring is present at the day 60 final time point. The FSG/3:1 mSTSG resulted in similar healing with less initial autografting requirements. Small wounds/scabs are present that are the result of biopsy punch sites. B) One representative wound is shown for the FSG/3:1 treatment group. Digital images at each day and the corresponding laser speckle images (LSI) throughout the experiment are shown. The heatmap scale is shown below in which blue represents low perfusion and red if highly perfused. Note that the perfusion in uninjured skin is a sky blue and as the wounds heal by day 60, the borders of the wound are no longer obvious. By day 60, the perfusion within the wound has returned to baseline levels indicated by the blue coloring.

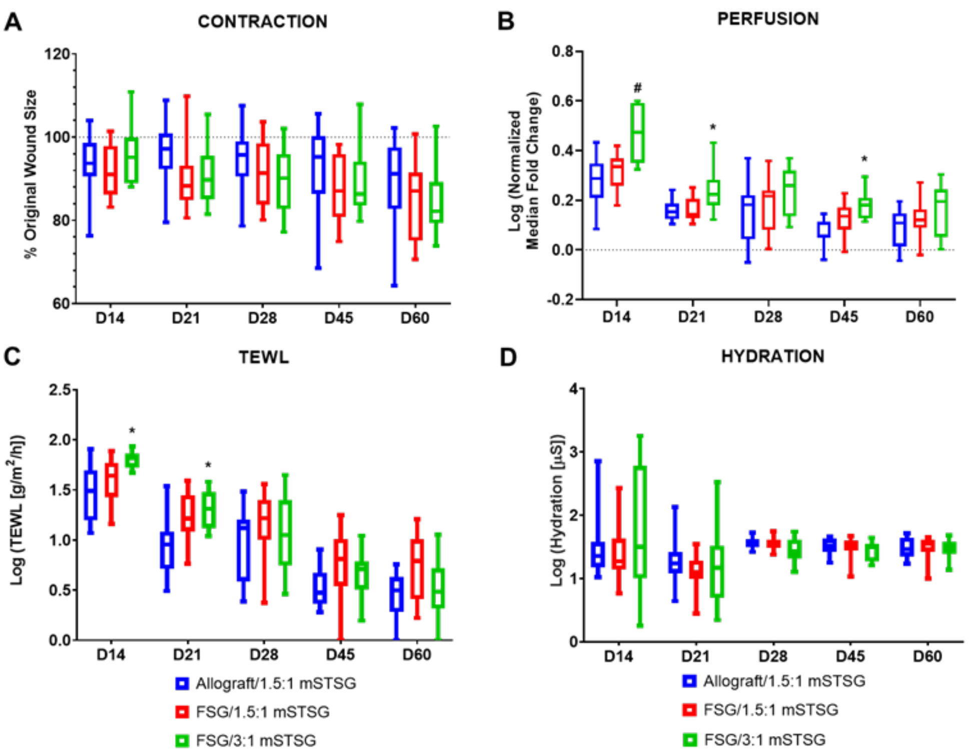

Supplemental Figure S2. Box and Whiskers Plot with 95% Confidence Intervals. A) Wound contraction was calculated by tracing the tattoos, comparing to the initial wound size, and normalizing to the growth of each animal. The dotted line represents the original wound size. Any reduction below that line is the result of the wounds contracting as they heal. No significant differences comparing the groups at each time point were detected by 2-way repeated measures ANOVA with Tukey's post-hoc test; B) Laser speckle imaging was used to measure the perfusion in the wounds. A fold change was calculated by wound/local background around each wound and normalized to the growth controls on each animal at the designated time point. The normalized median fold change was log transformed in order to pass the Shapiro-Wilk normality test. #= $p<0.05$ for FSG/3:1 vs. both groups and *= $p<0.05$ for FSG/3:1 vs. Allograft/1.5:1 treated wounds as determined by 2-way repeated measures ANOVA with Tukey's post-hoc test; C) TEWL measures the barrier properties of the epidermal layer of skin. The mean TEWL measurement was obtained by averaging 3 spots from each wound per time point. The mean was log transformed in order to pass the Shapiro-Wilk normality test. *= $p<0.01$ for FSG/3:1 vs. Allograft/1.5:1 treated wounds as determined by 2-way repeated measures ANOVA with Tukey's post-hoc test; D) The hydration measures the water content of the wounds. Hydration values of 5 spots within each wound were averaged. The mean was log transformed in order to pass the Shapiro-Wilk normality test. No significant differences comparing the groups at each time point were detected by 2-way repeated measures ANOVA with Tukey's post-hoc test. Results are shown as mean with 95% confidence intervals; N =10 (FSG/1.5:1 mSTSG); 12 (Allograft/1.5:1 mSTSG); 10 (FSG/3:1 mSTSG).

Epidermal Status

	FSG/3:1 mSTSG + FSG (N = 10)				
**Path Score**	**D14**	**D21**	**D28**	**D45**	**D60**
0 = no epidermis.	1	0	0	0	0
1= partial epidermis	2	0	0	0	0
2 = regenerating or hyperplastic with 100% coverage	6	7	1	0	0
3 = Normal epidermis across entire wound bed	1	3	9	10	10

Fibroplasia

	FSG/3:1 mSTSG + FSG (N = 10)				
**Path Score**	**D14**	**D21***	**D28**	**D45**	**D60**
0 - None	0	0	0	0	0
1 - Minimal	0	0	0	1	0
2 - Mild	2	1	5	6	3
3 - Moderate	7	7	4	3	6
4 - Marked	1	2	1	0	1

Foreign Material

	FSG/3:1 mSTSG + FSG (N = 10)				
**Path Score**	**D14**	**D21**	**D28**	**D45**	**D60**
0 - Absent	9	9	8	10	10
1 - Present	1	1	2	0	0

Angiogenesis

	FSG/3:1 mSTSG + FSG (N = 10)				
**Path Score**	**D14**	**D21**	**D28**	**D45**	**D60**
0 - None	3	6	9	9	10
1 - ≤50 vessels	6	4	1	1	0
2 - 51-100 vessels	1	0	0	0	0

Hemorrhage

	FSG/3:1 mSTSG + FSG (N = 10)				
**Path Score**	**D14**	**D21**	**D28**	**D45**	**D60**
0 - Absent	6	2	6	9	6
1 - Acute only	4	8	4	1	4

Hemorrhage Severity

	FSG/3:1 mSTSG + FSG (N = 10)				
**Path Score**	**D14**	**D21**	**D28**	**D45**	**D60**
0 - Absent	6	2	6	9	6
1 - Mild	3	8	3	1	4
2 - Moderate	1	0	0	0	0
3 - Severe	0	0	1	0	0

Neutrophils

	FSG/3:1 mSTSG + FSG (N = 10)				
**Path Score**	**D14**	**D21**	**D28**	**D45**	**D60**
0 - None	4	9	9	10	10
1 - Minimal number of inflammatory cells	5	1	1	0	0
2 - Mild number of inflammatory cells	1	0	0	0	0

Eosinophils

	FSG/3:1 mSTSG + FSG (N = 10)				
**Path Score**	**D14**	**D21**	**D28**	**D45**	**D60**
0 - None	4	5	8	10	9
1 - Minimal number of inflammatory cells	2	3	2	0	1
2 - Mild number of inflammatory cells	4	2	0	0	0

Lymphocytes

	FSG/3:1 mSTSG + FSG (N = 10)				
**Path Score**	**D14**	**D21**	**D28**	**D45**	**D60**
0 - None	2	4	5	8	4
1 - Minimal number of inflammatory cells	5	2	3	1	2
2 - Mild number of inflammatory cells	3	4	2	1	4

Macrophages

	FSG/3:1 mSTSG + FSG (N = 10)				
**Path Score**	**D14**	**D21**	**D28**	**D45**	**D60**
0 - None	3	5	6	8	5
1 - Minimal number of inflammatory cells	7	4	4	2	5
2 - Mild number of inflammatory cells	0	1	0	0	0

Supplemental **Figure S3. Histology Assessing Wound Bed After Grafting of FSG + 3:1 mSTSG/FSG Group.** A Veterinary Pathologist blinded to the groups scored the sections from day 14 thru day 60 for the stated parameters. For pathology scoring analysis comparing all three groups, the Kruskal–Wallis test with Dunn's multiple comparison test was used to test for differences in mean rank scores stratified by time. * = $P<0.05$ compared to Allograft treated wounds. No other differences were observed.
